# Efficacy of Robotic-Assisted Prostatectomy in Localized Prostate Cancer: A Systematic Review of Clinical Trials

**DOI:** 10.1155/2013/105651

**Published:** 2013-11-10

**Authors:** Carolina Sandoval Salinas, Andrés L. González Rangel, Juan G. Cataño Cataño, Juan C. Fuentes Pachón, Juan S. Castillo Londoño

**Affiliations:** ^1^Grupo de Investigación Clínica, Instituto Nacional de Cancerología, Calle 1 No. 9-85, Bogotá, DC 110321-012, Colombia; ^2^Centro Oncológico Javeriano, Hospital Universitario San Ignacio, Pontificia Universidad Javeriana, Carrera 7 No. 40-62, Bogotá, DC 110311-066, Colombia

## Abstract

*Background*. Radical prostatectomy is an effective treatment for clinically localized prostate cancer. The three approaches in current use have been extensively compared in observational studies, which have methodological limitations. *Objective*. To compare the efficacy and safety of three radical prostatectomy approaches in patients with localized prostate cancer: open, laparoscopic, and robotic-assisted laparoscopic surgery. *Materials and Methods*. A systematic review of the literature was carried out. Databases MEDLINE, EMBASE, LILACS, and CENTRAL were searched for randomized clinical trials that directly compared two or more radical prostatectomy approaches. Selection criteria, methodological rigor, and risk of bias were evaluated by two independent researchers using Cochrane Collaboration's tools. *Results*. Three trials were included. In one study, laparoscopic surgery was associated with fewer blood loss and transfusion rates than the open procedure, in spite of longer operating time. The other two trials compared laparoscopic and robotic-assisted surgery in which no differences in perioperative outcomes were detected. Nevertheless, robotic-assisted prostatectomy showed more favorable erectile function and urinary continence recovery. *Conclusion*. At the present time, no clear advantage can be attributed to any of the existing prostatectomy approaches in terms of oncologic outcomes. However, some differences in patient-related outcomes favor the newer methods. Larger trials are required.

## 1. Background

Radical prostatectomy constitutes a major cornerstone in the treatment of localized prostate cancer among patients whose life expectancy is greater than ten years [1]. Despite the fact that open prostatectomy is still the most widely used procedure in developing countries, state-of-the-art technologies such as laparoscopic and robotic-assisted laparoscopic prostatectomy offer minimally invasive alternatives to open surgery.

Although these surgical approaches are widely used, the quality of evidence that supports their efficacy is low. Most studies compare noncontemporary surgical series with retrospective data collection with short-term follow-up periods, thus increasing the risk of bias in their conclusions. Several reviews summarize these observational studies [2–14], showing very high heterogeneity, prognostic imbalance, and low adjustment for confounding among the uncontrolled studies, demonstrating the need for evidence provided by clinical trials, which reduce the high risk of bias and allow a more valid conclusion about which of the methods is most appropriate.

This study aims to describe comparative evidence derived from clinical trials on the three radical prostatectomy approaches currently used in the treatment of patients with localized prostate cancer: open, laparoscopic, and robotic-assisted laparoscopic surgery.

## 2. Materials and Methods

A systematic review of randomized clinical trials was performed. Studies that compared open radical, laparoscopic, or robotic-assisted prostatectomy for the treatment of localized prostate cancer (T1 or T2 stage) were included. A search was performed in the databases EMBASE (1974–October 2012), MEDLINE (1946–October 2012), LILACS (1982–October 2012), CENTRAL (1948–October 2012), and DARE (2002–October 2012), using the following strategy:

1. prostat$.mp.
2. Prostatectomy/
3. prostatectom$.tw.
4. (resect$ or excision or operat$ or remov$ or surg$).tw.
5. Surgical Procedures, Operative/ or surgery.fs.
6. (radical or complete$ or total or en bloc).tw.
7. (LRP or TLRP or RALRP or RAP or RRP or RPP or EERP or MIRP).tw.
8. heilbronn technique.tw.
9. ((2 or 3 or 4 or 5) and 6) or (7 or 8)
10. (open or incision$1 or laparotom$ or minilaparotom$).tw. or laparotomy/
11. (laparoscop$ or endoscop$ or celioscop$ or peritoneoscop$).tw. or Laparoscopy/
12. robot$.tw.
13. or/10–12
14. and/1, 9, 13.

Cochrane Collaboration's sensitivity and precision-maximizing filter for randomized trials (version 2008) were used [15]. No date limit was used; last update took place on December 4, 2012. Resulting references were screened based on titles and abstracts by two independent evaluators in accordance with the following criteria:

- clinical trials with randomized allocation that compare one of the surgical approaches being assessed, in men with localized prostate cancer;
- articles published in English or Spanish.

Full text of selected references was retrieved for review. Two independent evaluators extracted the information and assessed the methodological quality of the selected studies using Cochrane Collaboration's tool for evaluating risk of bias [15]. Extracted data included that for population and intervention characteristics, as well as early perioperative, postoperative (operating time, bleeding, transfusion rate, and complication rates), functional (recovery of urinary continence and sexual potency), and oncological (positive surgical margins and biochemical relapse) outcomes.

## 3. Results 

### 3.1. General Characteristics

Of the 576 references initially found, 217 were duplicates and 356 were ruled out after title and abstract review (81 were not related to the research issue, 169 evaluated interventions other than prostatectomy, 35 evaluated interventions in patients with benign prostatic hyperplasia, and 71 were nonrandomized clinical trials). Only three studies met the selection criteria. Out of the three clinical trials included, Guazzoni et al. evaluated the early perioperative and postoperative results of laparoscopic surgery compared to open surgery [16], and the remaining two compared laparoscopic surgery to robotic-assisted surgery [17, 18]. All of the studies included men under the age of 70 with clinically localized prostate cancer. Other clinical characteristics varied considerably among studies (Table 1). Given the clinical heterogeneity of the articles, no meta-analysis was executed.

### 3.2. Risk of Bias

**Figure 1:**
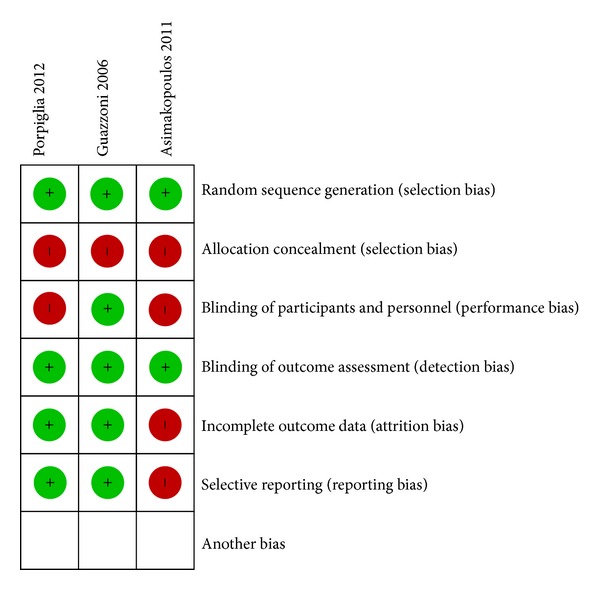
Risk of bias assessment of trials included in the review.

All trials had an adequate random sequence generation, but none of them performed allocation concealment. Only Guazzoni et al. reported blinding of personnel and patients. Blinding of objective outcomes was guaranteed in all studies. Evaluators considered that the trial of Asimakopoulos et al. had a high risk of both attrition and reporting bias (Figure 1).

### 3.3. Early Perioperative and Postoperative Outcomes

Guazzoni et al. found a significant difference in mean operation times favoring open prostatectomy (170 versus 235 minutes, *P* < 0.001). However, patients who underwent the laparoscopic procedure had lower intraoperative blood loss (853.3 versus 257.3 milliliters, *P* < 0.001), lower transfusion rates (45% versus 13.3%, *P* < 0.001, odds ratio 0.188, 95% CI 0.066–0.495), and higher frequency of hospital discharge at sixth postoperative day (86.6% versus 90%, *P* = 0.011) than the patients who underwent open surgery (Table 2).

No significant differences were observed among any of the early perioperative or postoperative outcomes (operation time, blood loss, transfusion rate, catheter time, days hospitalized, and rate of overall complications) in the comparison between laparoscopic and robotic-assisted surgeries.

### 3.4. Functional Outcomes

Both Asimakopoulos et al. and Porpiglia et al. reported an 11% greater urinary continence rate at 12 months in the robotic-assisted surgery arm, but this difference was only statistically significant in the latter (Table 2). Regarding recovery of sexual function at 12 months, both trials favored robotic-assisted surgery. This outcome was measured differently in both studies, and in Porpiglia et al., the comparison was made only in the nerve-sparring surgery subgroup. Asimakoupolus et al. reported that recovery of erectile function was faster among patients who underwent robotic-assisted prostatectomy (6.32 versus 2.27 months; *P* = 0.0001).

### 3.5. Oncological Outcomes

None of the three studies revealed significant differences in the percentage of positive surgical margins. Biochemical relapse-free survival at 12 months was similar between the laparoscopic surgery group and the robotic-assisted prostatectomy group (Table 2).

## 4. Discussion

A systematic review helps to underline the complexities related to the design and execution of controlled clinical trials. This review points out how difficult it is to adequately respond to questions concerning the application of novel surgical methods or modern technology, particularly in the field of cancer. The most serious barriers encountered in substantiating clinical trials for surgical interventions include indication bias, which is related to the surgical principle of selecting the best approach for each patient, availability of resources, and accumulation of a sufficient number of patients to allow a study to be performed [19]. Several authors have suggested that the synthesized results from well-executed observational studies, which are easier to do, can be compared to experimental studies with randomized allocation [20]; however, it should be understood that the quality of evidence regarding interventions differs greatly between the two designs, which in turn affects the strength of the recommendations derived from this evidence. Recent reviews identified many biases and unmanageable heterogeneity among the observational studies evaluated [14]. Although in this case the three trials provided better evidence, high risk of bias was also found from many design deficiencies: lack of allocation concealment, short follow-up periods, and small sample sizes.

Twenty-one years ago, the first radical laparoscopic prostatectomy was performed [21]. The first robotic platform appeared in the year 2000, thus improving the procedure through better visualization of the operating field, the provision of instruments adapted to the complex procedural technical necessities, and the enhancement of surgeon ergonomics. Retropubic open radical prostatectomy has been continually perfected during the past 20 years and now serves as the gold standard for the treatment of localized prostate cancer. Currently, radical prostatectomy is the most common robotic-assisted surgical procedure performed in the United States—a challenging fact when evaluating its possible introduction into a developing country such as Colombia [22].

Out of the three clinical trials included in this review, only one includes direct comparison with open surgery, a significant factor that hinders the reaching of valid conclusions, especially if we take into account that the most common comparator in the studies (nonrobotic-assisted laparoscopic prostatectomy) is not only the standard of care but also a technique clearly marked for obsolescence. 

There is very little of what we can ascertain at this point after having carried out this review, which is accountable to the quality of the evidence that is available. Nonetheless, we can ascertain that operation time for robotic-assisted laparoscopic prostatectomy has improved as surgeons have become more expert in its use: at the present time, it nearly reaches the same level as that for open prostatectomy carried out at high volume healthcare centers [23]. It is expected that postoperative pain will be lower with robotic-assisted surgery, an achievement that several patient series now suggest as possible [24]. 

The findings of the clinical trials are similar—in direction, but not in magnitude and statistical significance—to those reported in the systematic reviews of observational studies. Differences in results can be due to methodological limitations that affect the comparability of intervention groups in the observational studies. For instance, the comparison of noncontemporary cohorts has generally been erroneous because they weigh new robotic-assisted series against old open surgery series, placing the latter surgical approach at a disadvantage [5, 13], whereas in contemporary prospective series, it has been observed that no differences exist between open and robotic-assisted prostatectomy insofar as hospitalization time, hospital readmission, emergency room, or nonscheduled clinical visits are concerned [25]. In the trials, this risk of prognostic imbalance is lower thanks to their random assignment but is still present due to their small sample sizes.

Great variability exists in the published series [2, 3, 5, 6, 10, 11, 20] and in the clinical trials included in this review regarding secondary effects—such as urinary incontinence and erectile dysfunction—caused by the surgical procedure, therefore making a reliable comparison on this outcome practically impossible. Critical points which impede comparison include the nonstratification of patients by age, clinical status, and comorbidities, as well as lack of homogeneity in the definitions used and the different postoperative periods during which they were evaluated [19]. 

Regarding the oncologic outcomes, the trial's results confirmed the conclusion of the published series in which no surgical method prevailed over the other in the most frequently used endpoints such as surgical margins or biochemical recurrence-free survival [2, 7, 9].

## 5. Conclusions

There is no first-rate evidence that grants patent advantage to any novel prostatectomy method over the open approach in terms of oncological outcomes, surgical complications, or long-term secondary effects. However, when outcomes such as urinary continence and erectile function are the main concern, robotic-assisted prostatectomy shows an advantage. A decision should take into account aspects like patient's preference, surgeon's experience, and understanding of the procedure's requirements. When acquisition of any new medical device is under review by health centers, especially in countries and centers with limited resources, the genuine advantages, as well as the disadvantages, of the latest surgical method and its related technology—specifically, robotic-assisted prostatectomy—should be debated not only in economic terms but also in terms of organization, function, and individual quality of care.

## Figures and Tables

**Table 1:** Characteristics of studies included in the review.

Authors, year	Selection criteria	Groups	Characteristics
Guazzoni et al., 2006 [16]	*Inclusion*: men with prostate cancer <70 years, with clinically localized disease (cT1-cT2), serum PSA < 20 ng/mL, Gleason score ≤ 7 *Exclusion*: previous hormone blocking or previous prostate, bladder neck, urethra, or pelvic surgery; total prostate gland volume ≥ 60 mL	*Group* 1: radical retropubic prostatectomy (RRP), 60 patients *Group* 2: radical laparoscopic prostatectomy (RLP), 60 patients	Single surgeon trial: surgeon had 15 years of experience performing open prostatectomy and more than 150 laparoscopic procedures

Asimakopoulos et al., 2011 [17]	*Inclusion*: men with clinically localized prostate cancer (cT1-cT2), <70 years, serum PSA < 10 ng/mL, Gleason score ≤ 7, normal preoperative erectile function (IIEF-6 > 17), normal preoperative continence (IPSS) *Exclusion*: preoperative incontinence, moderate or severe erectile dysfunction (IIEF 6 < 17), neoadyuvant therapy, previous prostate, bladder neck or urethra surgery, positive MRI for extracapsular extension, nonbilateral nerve preservation	*Group* 1: radical laparoscopic prostatectomy (RLP), 64 patients *Group* 2: radical robotic-assisted laparoscopic prostatectomy (RRALP), 64 patients	(i) Single surgeon trial, with 900 conventional laparoscopic prostate resections and 300 robotic-assisted prostatectomies (ii) Continence was defined as absence of leakage or no need of use of protective pads (iii) Potency was defined as “capability of intercourse” and as an IIEF-6 scale score equal to or greater than 17, with or without the use of phosphodiesterase type 5 inhibitors (iv) Biochemical recurrence definition was not reported

Porpiglia et al., 2013 [18]	*Inclusion*: men with clinically localized prostate cancer according to TNM 2009 (T1-T2N0M0), remitted to institution for prostatectomy; between 40 and 70 *Exclusion*: previous radiotherapy and/or transurethral resection of the prostate gland	*Group* 1: radical laparoscopic prostatectomy (RLP), 64 patients *Group* 2: radical robotic-assisted laparoscopic prostatectomy (RRALP), 64 patients	(i) Single surgeon trial, with a reported experience of more than 600 laparoscopic prostatectomies and 100 robotic prostatectomies (ii) Continence was defined as the requirement of one or less safety pads per day (iii) Potency was defined as having an IIEF-5 scale score equal to or greater than 17 (iv) Biochemical recurrence was defined as PSA > 0.2 ng/mL

IIEF: International Index of Erectile Function; IPSS: International Prostate Symptom Score; MRI: magnetic resonance imaging.

**Table 2:** Comparison of outcomes among the three prostatectomy approaches.

Outcome	Guazzoni et al., 2006 [16]			Asimakopoulos et al., 2011 [17]			Porpiglia et al., 2013 [18]		
	PRP (*n* = 60)	LRP (*n* = 60)	*P* value	LRP (*n* = 60)	RALRP (*n* = 52)	*P* value	LRP (*n* = 60)	RALRP (*n* = 60)	*P* value
Lost to follow-up	0	0		4 (6.25%)	12 (18.75%)		0	0	
Operative time (min)	170 (34.2)	235 (49.9)	<0.001				138.1 (29.7)	147.6 (27.1)	0.068
Blood loss (mL)	853.3 (485)	257.3 (177)	<0.001				234.1 (150.1)	202 (124.0)	0.203
Transfusion	27 (45)*	8 (13.3)*	<0.001	3 (5)	0 (0)	0.1			
Catheterization time (days)				7.45 (2.3)	7.25 (2.7)	0.14	7.0 (0.5)	7.5 (3.9)	0.322
Catheter at 5th POP day	40 (66.6)	52 (86.6)	<0.001						
Hospital release at 6th POP day	52 (86.6)	54 (90)	0.011						
Hospitalization time (days)							4.6 (2.1)	4.8 (1.9)	0.5853
Postoperative complications									
Anastomotic leakage	20 (33)	8 (12)							
Acute urinary retention	1 (1.66)	1 (1.66)							
Fever	3 (5)	1 (1.66)							
Persistent lymphorrhea	5 (8.3)	4 (6.6)							
Rectal damage	0	1 (1.66)							
Total complications				5 (8)	8 (15)	0.24	7 (11.6)	10 (16.6)	0.433
Positive margins									
pT2	8 (18.25)	11 (24.4)	0.39	4 (7.7)	3 (7)	0.89	6/37 (16.2)	5/37 (13.5)	0.744
pT3	5 (31.24)	5 (33.3)	0.88	2 (25)	5 (55.6)	0.2	6/22 (27)	11/22 (50)	0.122
Total	13 (21.6)	16 (26)	0.28	6 (10)	8 (15.4)	0.39	12 (20)	16 (26.6)	0.388
Biochemical relapse				2 (3)	4(8)	0.3			
Biochemical relapse-free survival rate at 12 months							98%	92.5%	0.190
Recovery of urinary continence at 12 months				50 (83)	49 (94)	0.07	50 (83.30)	57 (95)	0.04
Urinary continence recovery time (months)				3.03 (2.92)	2.56 (4.21)	0.27			
Recovery of erectile function at 12 months				19 (32)	40 (77)	<0.0001	19 (54.2)^†^	28 (80)^†^	0.02
Sexual potency recovery (months)				6.32 (5.16)	2.37 (2.27)	0.0001			

RRP: radical retropubic prostatectomy; LRP: laparoscopic prostatectomy; RALRP: robotic-assisted laparoscopic radical prostatectomy; POP: postoperative.

Values expressed as numbers (percentage) or mean (standard deviation) accordingly. In some articles, only the number or percentage was reported.

*Autologous transfusion.

^†^Nerve-sparring surgery subgroup (*n* = 35 on each arm).
